# Sympathetic Responses to Noxious Stimulation of Muscle and Skin

**DOI:** 10.3389/fneur.2016.00109

**Published:** 2016-06-30

**Authors:** Alexander R. Burton, Azharuddin Fazalbhoy, Vaughan G. Macefield

**Affiliations:** ^1^School of Medicine, Western Sydney University, Sydney, NSW, Australia; ^2^School of Health and Biomedical Sciences, RMIT University, Bundoora, VIC, Australia; ^3^Neuroscience Research Australia, Sydney, NSW, Australia

**Keywords:** blood pressure, cutaneous pain, muscle pain, nociception, muscle sympathetic nerve activity, skin sympathetic nerve activity

## Abstract

Acute pain triggers adaptive physiological responses that serve as protective mechanisms that prevent continuing damage to tissues and cause the individual to react to remove or escape the painful stimulus. However, an extension of the pain response beyond signaling tissue damage and healing, such as in chronic pain states, serves no particular biological function; it is maladaptive. The increasing number of chronic pain sufferers is concerning, and the associated disease burden is putting healthcare systems around the world under significant pressure. The incapacitating effects of long-lasting pain are not just psychological – reflexes driven by nociceptors during the establishment of chronic pain may cause serious physiological consequences on regulation of other body systems. The sympathetic nervous system is inherently involved in a host of physiological responses evoked by noxious stimulation. Experimental animal and human models demonstrate a diverse array of heterogeneous reactions to nociception. The purpose of this review is to understand how pain affects the sympathetic nervous system by investigating the reflex cardiovascular and neural responses to acute pain and the long-lasting physiological responses to prolonged (tonic) pain. By observing the sympathetic responses to long-lasting pain, we can begin to understand the physiological consequences of long-term pain on cardiovascular regulation.

## Introduction

Pain serves a purpose. In normal physiology, it acts as a protective mechanism to prevent damage to tissues and causes the individual to react to remove or escape the painful stimulus (1). The sensory experience of pain has been described as “*the psychical adjunct of a protective reflex*” (2, 3). As pain is characteristically unpleasant, while other sensations are affectively neutral, the concept of sensation does not seem to encompass the experience of pain [Ref. (4) cited in Ref. (5)]. A more holistic approach to pain in humans is that of a homeostatic emotion reflecting an adverse condition in the body that requires a behavioral response (6). A direct offshoot of this behaviorist school of thought is that of adaptive behavioral responses to pain originating in different tissues: “*… noxious stimulation produces arousal, orientation, escape or immobilisation and help-seeking. Each of these is a behaviour that reflects a motivational demand”* (5).

While this homeostatic mechanism is crucial in maintaining the physiological integrity of the animal, an extension of the pain response beyond signaling tissue damage and healing, such as in chronic pain states, is of greater concern. The increasing number of chronic pain sufferers and the associated disease burden is putting healthcare systems around the world under significant pressure. Current research efforts are targeted toward understanding the mechanism behind the development of chronic pain and the transition from acute to chronic pain. It is widely accepted that chronic pain is established following activation of nociceptors in the initial stages, but can perpetuate to an ongoing experience of pain in the absence of ongoing noxious input. According to a recent position statement “*chronic pain may occur due to the persistent stimulation of nociceptors in areas of ongoing tissue*…[but]… *chronic pain persists long after the tissue damage that initially triggered its onset has resolved, and in some individuals, chronic pain can continue without ongoing tissue damage or preceding injury that can be detected with currently available diagnostic technology*” (7). Chronic pain is being increasingly recognized as a disease entity *per se*, rather than simply a symptom (8). The incapacitating effects of long-lasting pain are not just psychological – reflexes driven by nociceptors during the establishment of chronic pain may cause serious physiological consequences. Knowledge of these processes is crucial not just for devising complex future pain treatment approaches but also for preventing pain from becoming chronic, since nociceptive reflexes – through their actions on peripheral tissues via, for example, the sympathetic nervous system – may exacerbate ongoing pain.

The sympathetic nervous system is inherently involved in a host of physiological responses evoked by noxious stimulation. These include changes in blood flow to muscle and skin, as well as changes in blood pressure (BP), heart rate (HR), sweat release, and pupil diameter. Of these “fight or flight” responses, only HR and pupil diameter may include changes in parasympathetic outflow; the remainder are subserved by changes in sympathetic outflow exclusively (1). While the immediate, reflexly driven, physiological responses to acute pain are purposeful, teleological explanations for the prolongation of these responses in chronic pain are more obscure. Thus, understanding the physiological changes associated with long-lasting pain becomes important in understanding the transition from acute to chronic pain. It is plausible that the physiological reflexes seen in acute pain are also observed in chronic pain states, but also possible that reflexogenic changes are replaced by maladaptive physiological changes. The purpose of this review is to understand how pain affects the sympathetic nervous system by investigating the reflex cardiovascular and neural responses to acute pain and the long-lasting physiological responses to prolonged (tonic) pain.

## Animal Studies

The comprehensive studies by Jänig and colleagues, in which recordings were made from individual type-identified post-ganglionic sympathetic axons in the cat, have shown that noxious stimuli are capable of producing a differential response from cutaneous and muscle sympathetic neurones. Noxious stimulation of skin with mechanically damaging and thermal stimuli caused excitation in a small number of skin sympathetic neurones in the anesthetized cat; however, these responses were an exception to the rule and the majority of these neurones were inhibited by cutaneous noxious stimulation (9). Overall, most muscle vasoconstrictor neurones were excited during noxious stimulation of skin. This occurred in both anesthetized (9) and spinalized cats (10), thus suggesting the involvement of a spinal reflex: “*…the reflex pattern in muscular sympathetic fibres is the same on the spinal and on the brain stem level*” (11).

Sato and colleagues found that while rotation of the knee joint within the normal physiological working range did not produce any autonomic effects in anesthetized cats, the same movements caused increases in BP and HR when the knee joint was experimentally inflamed (12). Furthermore, they found that noxious rotation of knee joints (deep pain) caused an increase in BP and HR in both control and inflamed knee joints – with greater increases in BP and HR being seen with rotation of the inflamed joint. Given the increases in HR in response to torsion of inflamed knee joints seen in the Sato et al. (12) study were quite modest (8 ± 4 beats/min), and while they did not directly measure muscle sympathetic nerve activity (MSNA) – bursts of which cause vasoconstriction in muscle vascular beds and thereby increase total peripheral resistance – it may be inferred that the reported increases in BP were caused by increases in MSNA (and possibly splanchnic sympathetic nerve activity). Furthermore, it has also been shown that painful electrical stimulation produces excitatory effects upon the autonomic nervous system. Sciatic nerve stimulation, at intensities that activate A-delta (Group III) afferent fibers, is capable of causing increases in BP and HR in spontaneously hypertensive rats (13). Overall, this body of evidence demonstrates that nociception is capable of exerting excitatory effects on sympathetic outflow to muscle.

Some nociceptors are chemoreceptors sensitive to the concentration of irritant chemicals released into surrounding tissue by noxious thermal or mechanical stimuli, or reactive to exogenous chemicals that may penetrate the skin and bind to their sensory endings (14). Electrophysiological studies in experimental animals have shown that unmyelinated (group IV) and small-diameter (group III) nerve fibers in muscle respond both to the contraction and to the metabolic products of contraction (15–17). Moreover, activity in these afferents can be maintained by preventing release of the metabolic products; accordingly, these afferents are known as metaboreceptors (or ergoreceptors). Furthermore, lesion studies demonstrated that the reflex increase in sympathetically mediated vasoconstriction – the metaboreflex – requires the medulla (18); indeed, a somatosympathetic reflex can be demonstrated in the isolated brainstem–heart preparation (19). Electrophysiological evidence has revealed that group III and IV muscle afferents project to neurones in the rostral ventrolateral medulla (RVLM), the primary output nucleus for muscle vasoconstrictor drive (20), via the nucleus tractus solitarius and caudal ventrolateral medulla (CVLM) (21). Anatomically, it is expected that sustained noxious stimulation of muscle metaboreceptors can cause a supraspinally mediated increase in MSNA.

## Adaptive Behavioral Responses to Pain in Animals

In contrast to some of the aforementioned studies by Sato and colleagues, which suggest that noxious stimuli exert excitatory effects upon the sympathetic nervous system, it has been reported that pain originating in deep structures such as joints, muscle, and viscera may evoke profound decreases in BP and HR in rats (22). Keay and colleagues hypothesize that these different cardiovascular responses are components of an integrated response aimed at dealing effectively with the noxious stimulus. Stimulation of discrete parts of the brain supports this theory. Activation of a specific region within the midbrain, the lateral periaqueductal gray matter (lPAG), evokes an integrated response similar to that evoked by cutaneous pain – that is, an excitatory flight/fight response characterized by increases in BP and HR (23). Conversely, activation of the ventrolateral periaqueductal gray matter (vlPAG) evokes a response similar to that evoked by deep pain – a conservative/withdrawal response characterized by a decrease in BP and HR (24). This concept is further supported by studies demonstrating that cutaneous pain preferentially activates the lPAG, whereas deep pain (induced by intramuscular injection of 5% formalin solution) preferentially activates the vlPAG (25). These data suggest that the tissue from which pain originates may have a significant influence on both the behavioral and cardiovascular responses, and may be an additional factor to consider.

## Human Studies

Approximately half a century ago, Sir Thomas Lewis observed that pain originating in deep structures evoked very different behavioral and cardiovascular responses to pain originating in superficial structures (26). He observed that while pain originating in skin evoked “*a rise of pulse rate*” and a “*sense of invigoration*,” pain originating in deep structures evoked quiescence, a “*slowing of the pulse*” and “*falling of the blood pressure*.” Follow-up studies a decade later confirmed Lewis’ findings, where muscle pain was associated with a fall in BP and HR in awake human subjects (27). Interestingly, it appeared that a half-century hiatus passed without any further work being carried out in human subjects to determine whether pain originating in deep and superficial tissues produced differential effects on sympathetic activity until our group published in this area in 2009 (28, 29). Our lab has continued to explore the relationship between pain and sympathetic nervous system, shifting our focus from examining the effects of acute pain to that of tonic pain using a validated experimental model of pain.

For some time, the sympathetic nervous system has been considered to be involved in the chronic pain states formerly known as reflex sympathetic dystrophies (RSD), now labeled complex regional pain syndromes (CRPS). Subjects with CRPS often complain of a burning spontaneous pain in the distal part of the affected extremity. Presumed sympathetically mediated effects may cause the affected limb to be warm or cold; there may also be trophic changes (the skin wet or dry) and motor changes (30). There is evidence from human clinical studies supporting the idea that nociceptors develop catecholamine sensitivity after complete or partial nerve lesions; indeed, intraoperative stimulation of the sympathetic chain has been shown to induce increases of spontaneous pain in some patients with CRPS (30).

However, intraneural recordings of sympathetic nerve activity in human subjects have found no differences in sympathetic outflow to a painful limb compared to the contralateral non-painful limb, either in patients with CRPS suspected to be sympathetically maintained (31) or in healthy subjects suffering a unilateral experimental painful stimulus (32). Even if no regional change in sympathetic outflow to a painful limb has been demonstrated in man, it is feasible that physiological variations in sympathetic discharge may modulate nociceptive transmission, as suggested by studies on CRPS patients reporting augmented pain following the sympathoexcitation caused by arousal, forehead cooling (33), or whole-body cooling (34). Again, intraneural recording studies have not shown any relationship between the degree of sympathetic discharge and the level of perceived pain in CRPS patients (31, 35). While these studies have examined the effects of sympathetic activity in pathophysiological pain states – with the exception of our laboratory, relatively few groups have conducted intraneural sympathetic recordings in awake healthy human subjects using hypertonic saline to induce experimental muscle and skin pain, which better mimics clinical pain presentations.

Therefore, while the effects of short-lasting nociception on sympathetic outflow have been sufficiently investigated in a variety of both animal and human studies, the effects of long-lasting noxious stimulation on the regulation of sympathetic activity in humans are less understood. Indeed, undertaking such investigations may be more significant and relevant to understanding the role of the sympathetic nervous system in chronic pain states in humans. In order to examine the effects of tonic, long-lasting muscle pain on sympathetic outflow to skin and muscle, our group have used hypertonic saline and *infused* the solution through an intramuscular cannula over a 45-min period, which resulted in pain lasting for ~60 min (36–38).

## Experimental Pain and Microneurographic Studies in Humans

### Muscle Sympathetic Nerve Activity

Direct neural recordings from awake human subjects have demonstrated increases in MSNA and BP in response to various forms of noxious stimulation, such as instillation of soap solution in the eye, strong mechanical pressure on the nail (39), immersion of a hand in ice water (40), and mechanical pressure on the skin (41). The problem with these stimuli is that they are not very selective in terms of the source of noxious stimulation, with mechanical pressure on the nail and immersion of the hand in ice-cold water stimulating many types of tissue. Increases in MSNA can also be induced by pain associated with spontaneous or evoked cluster headaches, which likely have a vascular origin (42). Cutaneous noxious inputs have shown consistent results in animal studies, with the consensus being an increase in MSNA. Interestingly in humans, while the majority of painful stimuli can produce increases in MSNA, painful electrical stimulation to the skin causes a short-lasting decrease in MSNA (43, 44). Earlier studies by our group found that the effects of acute noxious stimulation of skin in awake human subjects, induced by subcutaneous injection of a bolus (0.2 ml) of hypertonic saline, mirror those found in studies in anesthetized animals – an increase in MSNA (28). Hypertonic saline specifically causes depolarization of C-fibers in the volume of tissue in which it is injected, allowing one to target a particular tissue and, hence, type of pain (45). Intramuscular injections qualitatively evoke a pain described as dull, diffuse, and poorly localized. By contrast, the quality of skin pain differs significantly and can be described as hot, sharp, burning, and clearly localized to a small area surrounding the injection site (28, 29, 45). Considerable use of the hypertonic saline model has been employed to characterize the sensory and motor effects involved in muscle pain. This is largely due to the quality of the induced pain being comparable to acute clinical muscle pain possessing both localized and referred pain characteristics (45).

In contrast to cutaneous noxious stimulation, the effects of deep muscular pain on MSNA have shown more diverse results in the literature. In agreement with the results of animal studies by Sato et al. (12), we found that – in most cases – deep short-lasting pain, induced by bolus (0.5 ml) intramuscular injection of hypertonic saline, generally caused an *increase* in MSNA in awake subjects (28). Indeed, these increases in MSNA – regardless of the source of noxious input (muscle or skin) – were associated with increases in BP and, as evidenced by the increase in HR, likely an increase in cardiac sympathetic drive. However, it must be noted that a minority of subjects displayed the inverse: a reduction in MSNA in response to short-lasting acute pain (28). Importantly, we also showed that the sympathetic responses to noxious stimulation of either muscle or skin depended on the integrity of supraspinal projections: intramuscular or subcutaneous injection of hypertonic saline into the paralyzed legs of individuals with spinal cord injury, who could not feel the pain, failed to generate any autonomic markers that would have indicated a spinal reflex; in other words, in awake human subjects, the sympathetic responses to pain are psychogenic (46).

What is less understood is the response of MSNA to long-lasting pain, which considering the clinical significance of chronic pain is more relevant and important to our understanding. The pain induced by bolus intramuscular or subcutaneous injections of hypertonic saline lasts only ~8 min, which is only long enough to study the acute effects of pain. In order to investigate the physiological consequences of long-term pain on cardiovascular regulation in healthy human volunteers, we decided to infuse hypertonic saline into the tibialis anterior muscle over ~45 min, which causes a deep dull ache – which often refers into the foot – that lasts about an hour. This is three times as long as that studied previously, in which an infusion of 20 min has been suggested to be a good clinical model for chronic muscle pain (47). We do not agree with this, but at the very least the approach may allow one to understand some of the initial physiological processes during the development of chronic pain. In our studies, we keep the pain at a constant level by adjusting the rate of infusion; subjects experienced a steady-state level of pain (tonic pain) rated 5–6/10 on a numerical rating scale (NRS).

**Figure 1:**
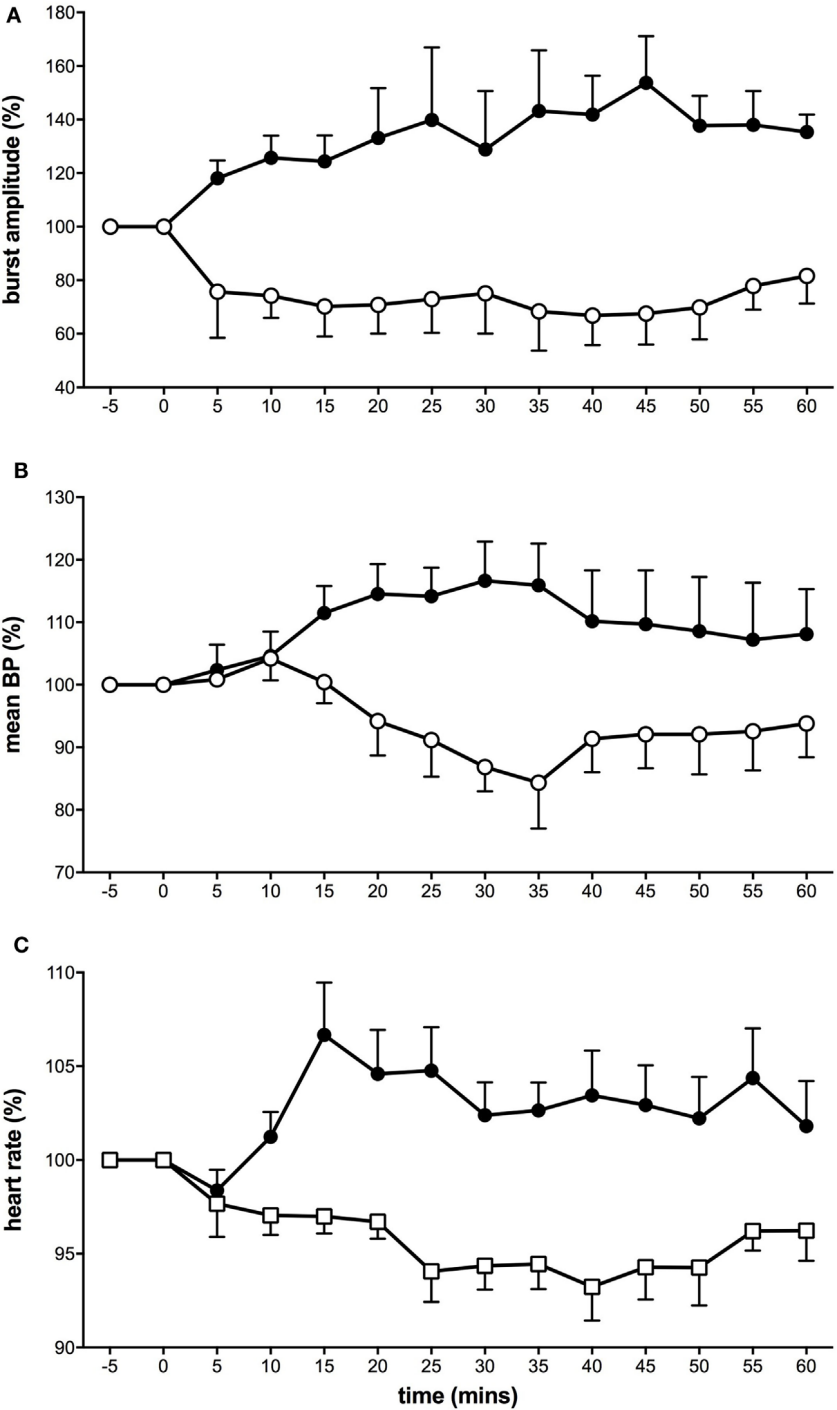
**Tonic muscle pain caused a dichotomy of responses in muscle sympathetic nerve activity (MSNA) (A), mean blood pressure (B), and heart rate (C)**. Subjects were divided into two groups: some who showed excitatory responses to muscle pain (solid black symbols) and others who showed depressed activity (open symbols) (36).

**Figure 2:**
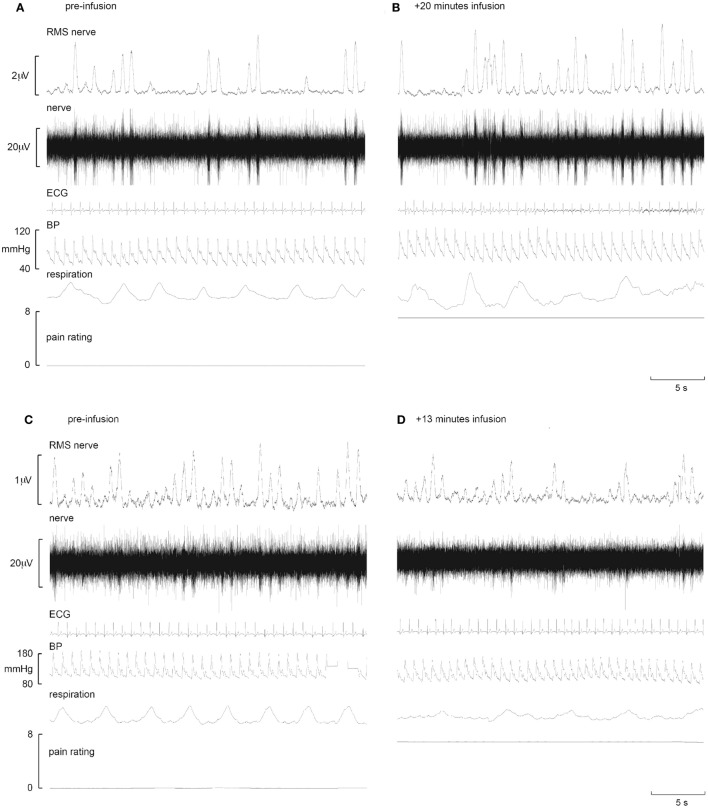
**Representative increases in neural and effector-organ responses (A,B) and decreases (C,D) in different subjects in response to tonic muscle pain**. Activity from one subject prior to tonic muscle pain **(A)** and 20 min post infusion **(B)** (ECG, electrocardiogram; BP, blood pressure; RMS, root mean square). MSNA from the common peroneal nerve shows little spontaneous activity pre-infusion **(A)**. Note the increases in burst frequency, burst amplitude, and BP during infusion of hypertonic saline **(B)**. By contrast, note the reduction of MSNA in another subject who showed reductions in MSNA burst frequency, burst amplitude, and BP when comparing baseline **(C)** to 13 min post infusion **(D)** [taken from Ref. (38)].

Interestingly, using this experimental model of tonic muscle pain, we showed that MSNA displayed two types of responses (Figure 1). Here, the proportion of subjects showing decreases in MSNA was greater in response to the long-lasting noxious stimulus than that we had observed following bolus injections. It would appear that these observations more closely support those reported by Lewis (26) where he noted “quiescence” or falls in BP in response to pain originating in deep structures. In about half of the subjects, MSNA burst amplitude increased, with parallel increases in BP, and HR (36). The other half of the subjects showed a decrease in MSNA, BP, and HR. Representative experimental records from these two contrasting responses are shown in Figure 2. These changes in MSNA matched the pain profile, beginning ~5 min after the start of the infusion of hypertonic saline and being maintained throughout the period of tonic muscle pain. Observing these parallel changes for the entire duration reveals that the sympathetic and cardiovascular responses are maintained by ongoing noxious stimulation. Moreover, the data suggest that the increase in MSNA was driving the increase in BP, which has been typically observed with essential hypertension (48–50). In some subjects, however, MSNA decreased but they still demonstrated an increase in BP during the pain. It is likely that in these subjects the fall in MSNA was caused by a baroreflex-mediated increase in BP. Conversely, a reduction in both BP and MSNA could be the result of a nociceptor-driven withdrawal of MSNA.

Heart rate variability (HRV) is an often over-interpreted physiological marker purportedly reflecting the relative contributions of sympathetic and parasympathetic (vagal) outflow to cardiac control (51). Spectral decomposition of the ECG reveals two primary bands: the high-frequency (HF) domain and the low-frequency (LF) domain. The HF band (0.15–0.4 Hz) reflects vagal cardiac control (52), while the LF band (0.04–0.15 Hz) has been suggested to represent (primarily) sympathetic cardiac activation (53). However, evidence supporting the cardiac sympathetic origin of the LF component is not very strong, and recent work suggests that the LF band does not reflect cardiac sympathetic outflow *per se* but simply modulation of cardiac autonomic drive via the baroreflexes (54). In our first tonic, pain study differences in baseline HRV data suggested that the increasing MSNA group had a greater LF power at baseline and during pain assessed at three separate time intervals (36). However, when the study was expanded to include a larger number of participants (*n* = 50) this was shown not to hold, and there was no difference between any HRV parameters when comparing the increasing or decreasing group (55).

**Figure 3:**
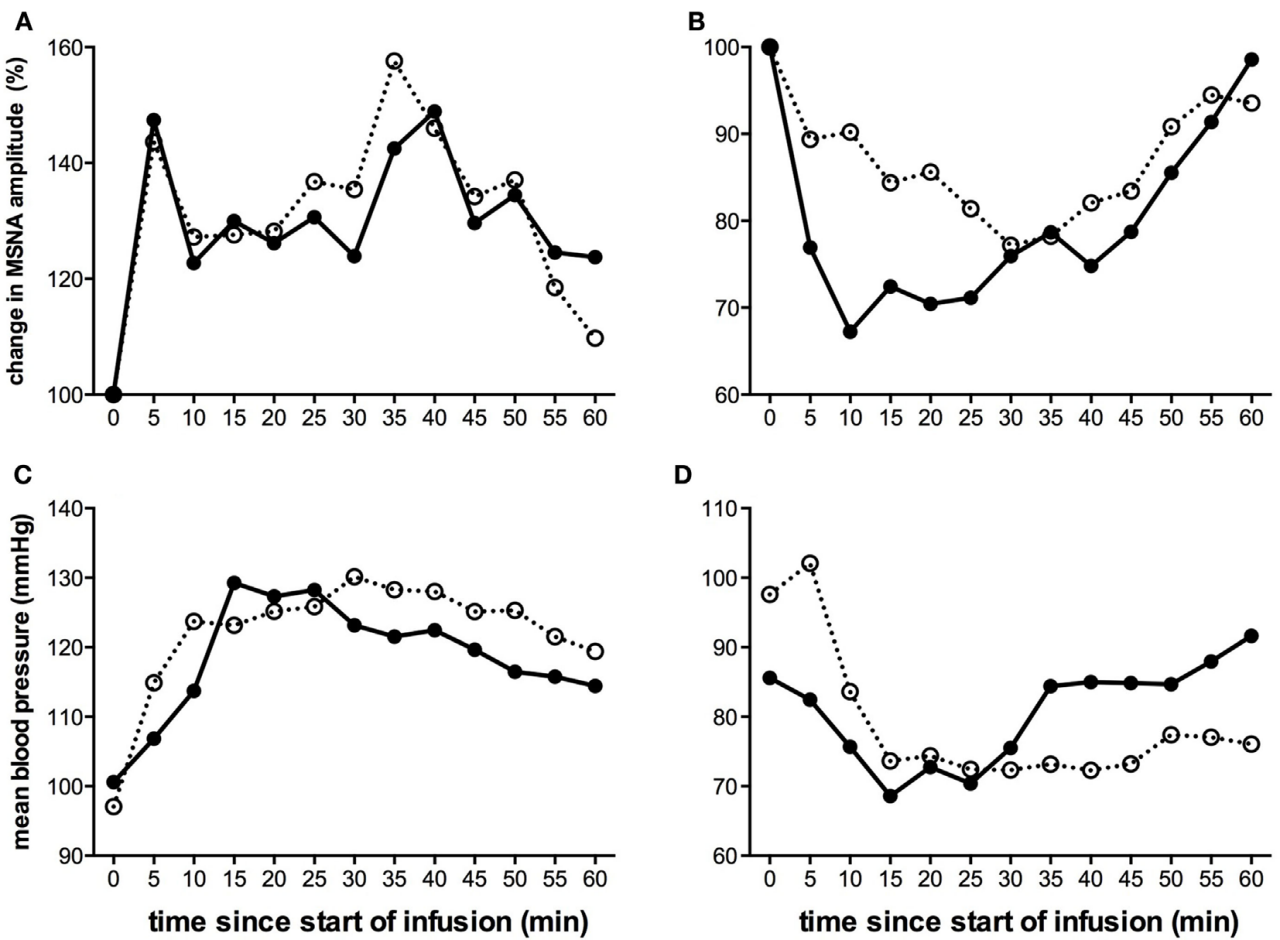
**Changes in mean MSNA burst amplitude (A,B) and mean blood pressure (C,D) during tonic muscle pain induced by intramuscular infusion of hypertonic saline for two subjects (A,C) and (B,D) during two separate recording sessions (open and closed symbols)**. The subject illustrated on the left **(A,C)** exhibited a sustained increase in MSNA and BP during the course of infusion, while the subject illustrated on the right **(B,D)** showed a decrease. For both subjects, the direction of the changes was the same in both recording sessions [taken from Ref. (38)].

Given that the level of MSNA is consistent in a given individual on a day-to-day basis (56), we predicted that the pattern of sympathetic response to tonic muscle pain would be identical across time. This was born out in a second study, in which we repeated the experiments in the same individuals after 3–27 weeks. In the majority of subjects, there was a consistent change in the direction of MSNA (Figure 3), which either increased in both sessions or decreased in both sessions; the changes in BP and HR were also the same in both recording sessions (38). We reasoned that these consistent changes infer that there are inherent characteristics of an individual that could potentially predict whether they would increase or decrease in MSNA during long-lasting pain. However, in a large study of 50 subjects, we found no differences in baseline levels of MSNA, BP, HR, HRV, age, or body-mass index that could account for these differences (55).

Gender was an important factor to consider, given that it has been shown that sex differences exist in certain aspects of cardiovascular control. For instance, while the control and sensitivity of the sympathetic baroreflex is comparable in both young men and women (57–59), it is known that that the increases in MSNA in response to head-up tilt differ between males and females (60). However, we showed that there was no effect of gender, as assessed in 25 males and 25 females: there was no difference in the *propensity* of males or females to exhibit an increase or decrease in MSNA during tonic muscle pain (55).

Given that a threatening stimulus, such as a strong noxious stimulus, would be expected to increase sympathetic outflow to many tissues as part of the fight or flight response, we speculated that subjects showing higher anxiety levels would show increases in MSNA during tonic muscle pain, while those who are better able to cope show decreases. However, in a recent psychometric study of 66 subjects, we showed that there were no differences in anxiety scores, assessed by the *State and Trait Inventory*, in the group of subjects in whom MSNA increased during tonic muscle pain and the group in whom MSNA decreased (61). Moreover, there were no differences in attitudes to pain between the two groups, as assessed using the *Vigilance and Awareness Questionnaire*, the *Pain Anxiety Symptoms Scale*, and the *Pain Catastrophizing Scale* (61). So, given the absence of baseline physiological or psychological differences between the two groups of subjects, we still do not know why there are two patterns of sympathetic response during tonic muscle pain.

### Skin Sympathetic Nerve Activity

**Figure 4:**
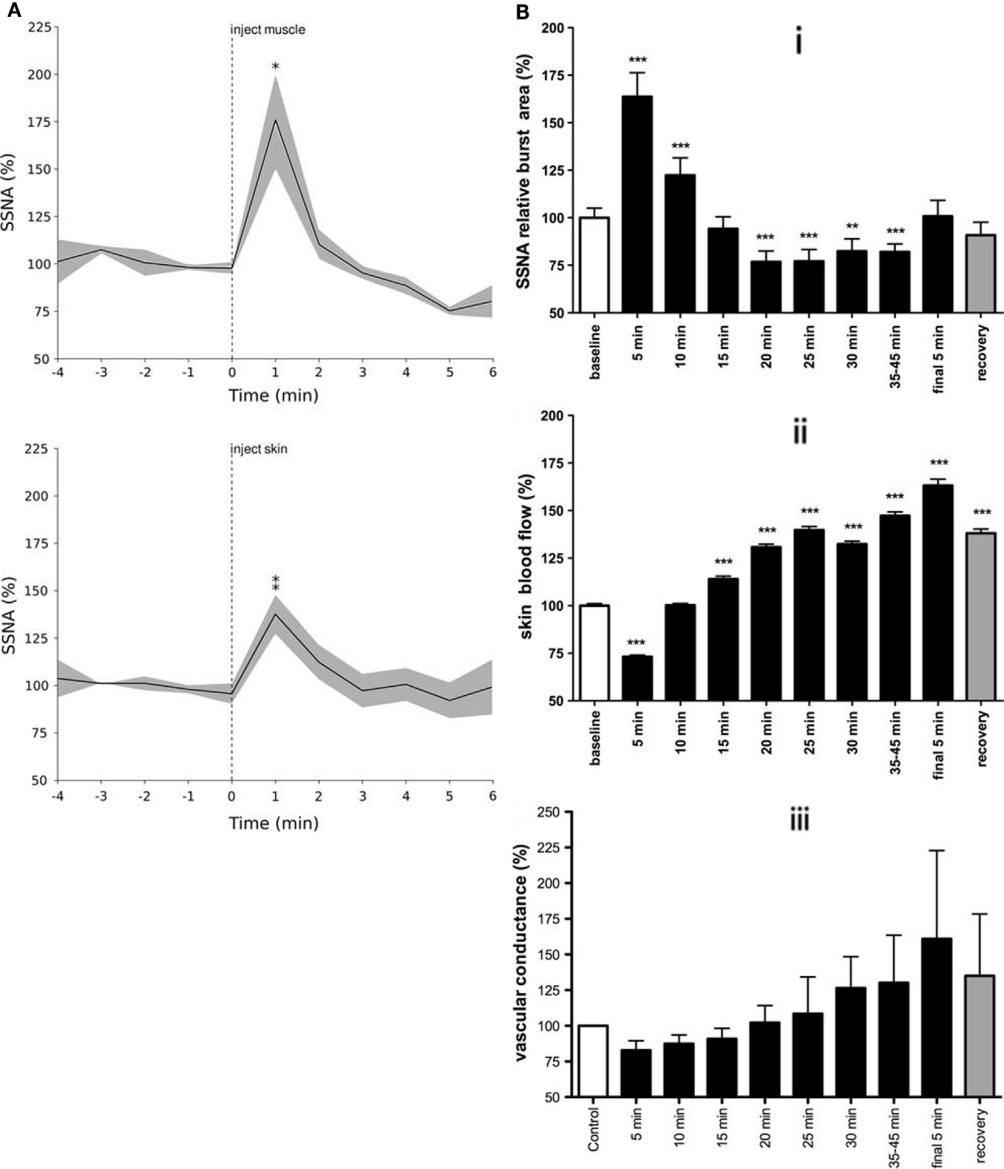
**Skin sympathetic nerve activity increased around the onset of pain for both bolus injections of hypertonic saline (A) and infusions (B)**. Note the biphasic response seen in B in response to tonic muscle pain: a prolonged decrease in SSNA following an initial surge in activity (i), with resultant changes in skin blood flow (ii), and vascular conductance (iii). This biphasic response is presumably related to the arousal component of pain, characterized by an increase in SSNA with concomitant decreases in skin blood flow followed by decreases in SSNA and increased skin blood flow [modified from Ref. (29, 37)].

Specific work regarding the influence of mental or emotional stress on skin sympathetic nerve activity (SSNA) is sparse (62). It would appear from the literature that the majority of studies examining the relationship between painful stimuli and SSNA have largely been used for the purpose of identifying SSNA (63–65). Depending on the duration of the painful stimulus, our group found distinct responses when examining the change in SSNA over time. However, unlike the general sustained responses seen in MSNA, the increases in SSNA were brief and occurred irrespective of whether the source of the pain was muscle or skin. Figure 4 shows that transient increases in SSNA occurred irrespective of whether the noxious stimuli were short-lasting (~4–5 min) bolus injections of hypertonic saline (29) or 45-min infusions in which pain lasted about an hour (37). The noteworthy difference between bolus injections and infusions is that the latter exhibited a biphasic response: a prolonged decrease in SSNA (and increase in skin blood flow), which followed the initial surge in SSNA (and resultant decrease in skin blood flow and increases in sweat release).

Skin sympathetic nerve activity showed a transient increase in activity around the onset of pain (29). Since the painful stimuli lasted much longer than these transient neural and effector-organ responses, it is highly likely that cardiac and skin autonomic responses are related to the initial onset and sudden surge in pain level, essentially reflecting an arousal or alerting response. The concept of nociception acting as an arousal stimulus is supported by the fact that the increase in SSNA occurred slightly *before* the subjective onset of pain. This suggests that the neural response to a noxious stimulus is associated with a transient change in homeostasis – a transition from no pain to that of pain. Unlike animal studies, it remains unclear whether cutaneous vasoconstrictor neurones in humans are reflexly inhibited during noxious stimulation of the skin (66), and whether the biphasic response to tonic muscle pain merely involves passive withdrawal of activity following the initial increase (37). As noted above, the fact that there were no signs of sympathetic activation (e.g., cutaneous vasoconstriction or sweat release) in individuals with spinal cord injury, who could not feel a painful stimulus delivered to their paralyzed legs, supports our conclusion that the initial increase in SSNA depends on the integrity of supraspinal pathways (46). In other words, the increase in SSNA reflects an affective response to the perception of a painful stimulus.

## General Discussion of Findings

Our studies have also raised other questions, which invite further investigation. One novel finding in the Burton et al. (29) study is that the direction of skin blood flow responses to nociception depended on gender, with males exhibiting decreases in skin blood flow and females showing increases. Cooke et al. (67) found that mental arithmetic and deep inspiration, maneuvers that causes increases in cutaneous sympathetic outflow, caused reductions in hand and skin blood flow in men but increases in women (67, 68). Cooke et al. (67) further demonstrated that the responses to mental arithmetic were dependent on the prevailing level of sympathetic tone to resistance vessels by using whole-body heating or cooling to increase or decrease central sympathetic outflow to the hand (68). These effects are comparable to our findings, where we demonstrated for the first time that gender may play a role in determining how skin blood flow changes following acute noxious stimulation. However, studying skin blood flow changes during long-lasting muscle pain did not show any such difference between genders.

It is evident from our studies that certain subjects will show increases in MSNA during intramuscular infusion of hypertonic saline that are sustained as long as the painful stimulus is maintained. Given that a proportion of post-surgical patients will go on to develop chronic pain while others do not (69), this raises interesting questions about how certain patients recover and others have ongoing pain even when tissue healing has clearly taken place. Keay and Bandler (70) discuss the idea of pain being categorized into escapable and inescapable. Since noxious events signal the necessity to take action (71), once nociceptors are stimulated and signals are received at the cortex, higher sensory processing determines the level of threat, evoking an appropriate integrated response. Logically speaking, pain originating in deeper structures would be inescapable, and pain originating from superficial structures would be escapable.

It is intriguing then to see a dichotomy of responses to intramuscular infusion, a method of nociceptor stimulation in deep tissues, that half of our subjects consistently responding with increasing sympathetic drive, which aligns more with the response typically associated with escapable pain. This finding is bizarre, on the one hand, yet exciting, on the other, as it could be a predictor of a subgroup of patients who may potentially develop chronic pain after sustaining injury due to the contribution of sustained increases in sympathetic drive.

It is plausible that the evoked response and changes in sympathetic drive, which we have demonstrated, outlast the noxious input and may contribute to the development of chronic pain and associated cardiovascular comorbidities. These series of experiments are in no way designed to definitively answer questions on the development of chronic pain. However, they certainly provide insightful evidence into the cardiovascular consequences of long-lasting pain.

## Implications

Unlike the short-lasting pain we had previously induced by bolus injections (28, 29), we believe the physiological responses to tonic pain will more closely replicate episodes during which chronic pain patients are suffering and coping with their pain. Indeed, persistent deep pain is characterized by a passive coping response (70) and tonic muscle pain lasting only 20 min has been used as a model for chronic musculoskeletal pain. This method of inducing muscle pain offers the advantage of allowing a controlled investigation into how pain may modulate MSNA, BP, and HR. Conversely – assuming everything else is equal – one would *need to know the level of MSNA in a person prior to the development of chronic pain in order to interpret any changes in muscle sympathetic outflow*. There is only one study to have reported changes in sympathetic nerve activity during chronic pain: in a single patient with chronic regional pain syndrome (CRPS), suspected to be sympathetically maintained because of the marked cutaneous vasoconstriction, there was no difference in sympathetic outflow to the painful limb compared to the contralateral non-painful limb (35). Perhaps this is because both limbs were affected equally – in most instances, sympathetic outflow is distributed symmetrically to the left and right limbs. We postulate that a person who consistently exhibited increases in MSNA, BP, and HR during experimental muscle pain may – if he or she developed chronic pain from an injury in the future – go on to develop high BP. Interestingly, in a retrospective study, Bruehl et al. (69) showed that patients with *post-surgical chronic pain* have approximately twice the prevalence of clinical hypertension (39%) than patients without pain (21%). Why this should be is not known.

## Author Contributions

AB completed majority of the body of the review in all sections of manuscript. AF contributed to body of review particularly relating to all information regarding studies post 2009. Contributed all sections regarding sustained (infused) hypertonic saline pain models used in experiments. Intellectual contribution, clarification, and editing across entire manuscript. VM integrated contributions of AB and AF, and authored context for review and relevance to broader issues regarding pain. Major intellectual contribution and clarification of body of text, abstract, implications section, and general discussion section.

## Conflict of Interest Statement

The authors declare that the research was conducted in the absence of any commercial or financial relationships that could be construed as a potential conflict of interest.
